# A new approach to define the optimal immunization strategy against pneumococcal disease: the example of Canada

**DOI:** 10.1017/S0950268825000305

**Published:** 2025-03-19

**Authors:** Philippe De Wals

**Affiliations:** 1 Department of Social and Preventive Medicine, Laval University, Quebec City, QC, Canada; 2 Clinical Research Center, Sherbrooke University Hospital Center, Sherbrooke, QC, Canada; 3 Quebec National Public Health Institute, Quebec City, QC, Canada

**Keywords:** pneumococcal disease, pneumococcal conjugate vaccine, immunization schedule, immunization program, prevention strategy

## Abstract

New-generation pneumococcal conjugate vaccines (PCVs) are available to replace PCV-13 for childhood and adult immunization. Besides cost-effectiveness evaluations which have highly variable results, the comparative immunogenicity of these new vaccines (PCV15, PCV20, PCV21) and their coverage of invasive pneumococcal disease (IPD) and carriage strains in different age-groups should be regarded as well as the antibody susceptibility, antibiotic resistance, invasiveness and virulence of serotypes included in each vaccine. Based on the Canadian experience, these topics are discussed. The optimal strategy would be a 2+1 PCV20 schedule for children, PCV21 for elderly adults and a dual PCV20+PCV21 schedule for adults at very high IPD risk. Shifting from PCV-13 to PCV-15 for children entails a risk of increased IPD incidence in adults because additional serotypes are of low virulence and could be replaced by more invasive and virulent serotypes. This risk can be reasonably excluded if PCV-20 replaces PCV-13 as the former covers additional serotypes being highly invasive and virulent. It is recognized that off-label use of PCV-20 according to a 2+1 schedule could be problematic for some jurisdictions as this is not authorized in all countries. In Canada, however, the 2+1 PCV20 schedule was authorized based on the same dataset submitted elsewhere.

## Introduction

The 13-valent pneumococcal conjugate vaccine (PCV-13) is widely used worldwide for the prevention of pneumococcal diseases both in children and adults [1]. In Canada as in many European countries and the USA, a 15-valent vaccine (PCV-15) and another 20-valent vaccine (PCV-20) have been authorized for use in children and adults [2, 3]. A notable peculiarity is that PCV-20 was authorized for children on a 2 + 1-dose schedule by Health Canada [3]. Based on the same dataset, PCV-20 was authorized by the European Medicine Agency according to a 3 + 1 schedule, rather than a 2 + 1 schedule [4]. In the USA, all PCVs are authorized for children according to a 3 + 1 schedule only [5]. A 21-valent vaccine (PCV-21) that was specifically designed to target *Streptococcus pneumoniae (Sp)* serotypes observed in invasive pneumococcal disease (IPD) in adults in a context of PCV use in children has been authorized in the US and Canada [6, 7]. Traditionally, national recommendations on the use of PCVs focus on immunogenicity, safety, potential impact, and cost-effectiveness considerations [8]. Results of cost-effectiveness evaluations of PCVs vary widely due to uncertainties regarding the nature and intensity of serotype replacement, as well as the value of key input parameters such as the *Sp* attributable fraction and serotype distribution among non-invasive outcomes [9, 10]. A full assessment of the potential usefulness of any PCV should, however, capture other characteristics of targeted serotypes including their susceptibility to circulating antibodies, antimicrobial resistance, invasiveness, and virulence. Based on the Canadian experience, the objective of this manuscript is to compare the three new-generation PCVs (PCV-15, PCV-20, and PCV-21) in terms of invasive pneumococcal disease (IPD) and carriage strain coverage, antibody susceptibility, antibiotic resistance, invasiveness, and virulence of vaccine-serotypes, as well as the relative immunogenicity of these three vaccines. This new approach would help expert committees and public health authorities in other jurisdictions on the optimal immunization strategy for controlling pneumococcal disease.

## Serotype composition of vaccines

PCV-13 contains 13 serotypes (1, 3, 4, 5, 6A, 6B, 7F, 9V, 14, 18C, 19A, 19F, and 23F). Serotype 6C is also covered through cross-protection provided by the 6A component [11]. PCV15 adds two serotypes (22F and 33F) and PCV-20 adds five non-PCV15 serotypes (8, 10A, 11A, 12F, and 15C). Serotypes 15B and 15C should be considered as a single entity (15B/C) and cross-protective antibodies are generated by each of the two components [12, 13]. PCV-21 was designed to target frequent serotypes in adults in a context of PCV use in children: 11 serotypes also in PCV-20 (3, 6A, 7F, 8, 10A, 11A, 12F, 15C, 19A, 22F, and 33F) and 10 specific serotypes (9 N, 15A, 16F, 17F, 20, 23A, 23B, 24F, 31, and 35B). Together, PCV-20 and PCV-21 cover 31 serotypes (30 vaccine-serotypes + 6C). These two vaccines are highly complementary and their combined use in a specific population would provide optimal broad protection.

## Coverage of invasive pneumococcal disease strains

In Canada, the National Microbiology Laboratory (NML) in Winnipeg, Manitoba, affiliated with the Public Health Agency of Canada has the mandate of IPD surveillance [14, 15]. Invasive *Sp* isolates and biological specimens are transmitted from provincial and territorial public health laboratories for confirmation of bacteriological diagnosis, serotyping, and analysis of the antimicrobial resistance profile. For the province of Quebec, selected culture-negative biological specimens and *Sp* isolates are transmitted along with aggregated data on all IPD cases reported to the *Laboratoire de Santé publique du Québec (LSPQ)* affiliated with the *Institut national de Santé publique du Québec (INSPQ).*

During the period 2018–2022, an important variation in the total number of culture-positive IPD cases was observed (3340 cases in 2018, 3690 in 2019, 2122 in 2020, 2008 in 2021, and 3846 in 2022), a consequence of the many disturbances associated with the COVID-19 pandemic and a surge in respiratory viral infections starting in the fall of 2021 when COVID-19 control measures were lifted [16].

The serotype distribution of IPD cases with a culture originating from a normally sterile site or liquid reported in the years 2018–2022 and with information on age is shown in Table 1. The proportion of IPD cases attributable to PCV-13-serotypes was higher in the 5–64 years age group (42%) than in younger and older age groups (24% and 30%, respectively). The two additional serotypes included in PCV-15 accounted for about 11% of cases in all age groups. The fraction of IPD cases attributable to the five additional serotypes included in PCV-20 decreased with age: 25% in children <5 years, 20% in 5 to 64-year-olds, and 15% in elderly adults. In contrast, the proportion of cases caused by the 10 serotypes specific to PCV-21 was 24% in children and middle-aged adults and 33% in elderly adults. During the study years, non-vaccine types (NVT) represented about 7% of all IPD cases.

**Table 1.** The proportion of IPD cases reported in Canada in 2018–2022 belonging to *Streptococcus pneumoniae* serotypes included in 13-valent, 15-valent, 20-valent, and 21-valent pneumococcal conjugate vaccines and the proportion of cases showing resistance to at least one or three antimicrobials

Sérotype category	Number (%) of IPD cases			Proportion (No/No. tested and %) of serotype resistant to antimicrobial classes^b^	
	< 5 year-olds	5–64 year-olds	 *65 year-olds*	At least one	Three or more
PCV–13^c^	262 (24.2%)	3544 (42.0%)	1591 (30.2%)	697/2603 (26.8%)	324/2603 (12.4%)
PCV–15 non-PCV–13	173 (16.0%)	706 (8.4%)	702 (13.3%)	488/827 (59.0%)	26/827 (3.1%)
22F	124 (11.5%)	527 (6.2%)	542 (10.3%)	294/606 (48.5%)	18/606 (3.0%)
33F	49 (4.5%)	179 (2.1%)	160 (3.0%)	194/221 (87.8%)	8/221 (3.6%)
PCV–20 non-PCV–15	266 (24.6%)	1718 (20.3%)	769 (14.6%)	494/1492 (33.1%)	55/1492 (3.7%)
8	16 (1.5%)	626 (7.4%)	202 (3.8%)	11/458 (2.4%)	0
10A	45 (4.2%)	116 (1.4%)	93 (1.8%)	24/132 (18.2%)	0
11A	36 (3.3%)	203 (2.4%)	214 (4.1%)	113/261 (43.3%)	15/261 (5.7%)
12F	9 (0.8%)	623 (7.4%)	122 (2.3%)	263/382 (68.8%)	25/382 (6.5%)
15B/C^a^	160 (14.8%)	150 (1.8%)	138 (2.6%)	83/259 (32.0%)	15/259 (5.8%)
PCV–21 non-PCV–20	259 (23.9%)	2050 (24.3%)	1750 (33.2%)	467/2050 (22.8%)	233/2050 (11.4%)
9 N	20 (1.8%)	541 (6.4%)	344 (6.5%)	46/447 (10.3%)	10/447 (2.2%)
15A	44 (4.1%)	204 (2.4%)	293 (5.6%)	122/232 (52.6%)	100/232 (43.1%)
16F	23 (2.1%)	166 (2.0%)	178 (3.4%)	11/184 (6.0%)	7/184 (3.8%)
17F	5 (0.5%)	62 (0.7%)	57 (1.1%)	13/83 (15.7%)	3/83 (3.6%)
20	14 (1.3%)	463 (5.5%)	145 (2.8%)	20/320 (6.3%)	12/320 (3.8%)
23A	30 (2.8%)	198 (2.3%)	239 (4.5%)	88/240 (36.7%)	57/240 (23.8%)
23B	83 (7.7%)	192 (2.3%)	182 (3.5%)	37/225 (16.4%)	6/225 (2.7%)
24F	10 (0.9%)	17 (0.2%)	21 (0.4%)	25/32 (78.1%)	14/32 (43.8%)
31	4 (0.4%)	98 (1.2%)	112 (2.1%)	8/118 (6.8%)	0
35B	26 (2.4%)	109 (1.3%)	179 (3.4%)	97/169 (57.4%)	24/169 (14.2%)
NVT	122 (11.3%)	428 (5.1%)	456 (8.7%)	197/586 (28.1%)	44/586 (6.3%)
Total	1082 (100%)	8446 (100%)	5268 (100%)	2343/7558 (31.0%)	682/7758 (8.8%)

a Serotypes 15B and 15C are combined into one category.

b Number of isolates with completed antimicrobial resistance testing is lower than the total submitted to the laboratory.

c Including ST-6C. NVT = Non-Vaccine-Type.

Trends in the distribution of serotypes were observed in recent years, with an increase in the proportion of serotypes covered by PCV-20 and a decrease in the proportion covered by PCV-21, these two opposite trends being more pronounced in the 5–64 years age groups than in elderly adults and children (not shown in the table). Currently, serotype distribution across age groups suggests that PCV-20 is particularly suited for children, while PCV-21 is the best choice for elderly adults, especially if PCV-20 is used in children, generating a herd effect and serotype replacement, thereby increasing the incidence of PCV-21-specific serotypes in adults.

## Coverage of strains in asymptomatic carriers

Asymptomatic carriers play a key role in the transmission of *Sp* and the epidemiology of IPD [17]. The prevalence of *Sp* carriage in the population is influenced by age, with children of kindergarten age (2–5 years) having the highest rates [18, 19]. Carriage density of is a key factor in the transmission and is highest in young children [20]. The immunization of children with PCVs has been associated with a reduction in the proportion of carriers of vaccine serotypes and an increase in the proportion of non-vaccine serotypes, the replacement being generally complete or sometimes partial [21].

Information on the dynamic of carriage in Canada is limited [22]. In the United Kingdom, two large surveys were conducted in the context of a mature PCV-13 programme based on a 2 + 1 schedule as in Canada [23, 24]. In children 1–4 years of age, the proportion of strains identified in carriers belonging to PCV-15-specific serotypes (22F and 33], were respectively 7% and 6% in the two surveys, the proportion being respectively 36% and 27% for PCV-20-specific serotypes (8, 10A, 11A, 12F, 15B/C, 22F, and 33F) [23, 24]. These observations suggest that the magnitude of replacement could be higher if PCV-20 replaces PCV-13 than if PCV-15 replaces PCV-13. The consequence of this replacement would, however, be determined by the invasiveness and virulence of the serotypes that will occupy an empty ecological niche.

## Correlates of protection for invasive pneumococcal disease

PCV-13 serotype-specific correlates of protection for infants have been estimated by linking serotype-specific IgG concentration distributions after two priming doses in randomized clinical trials (RCTs) and serotype-specific vaccine effectiveness (VE) estimates using the indirect cohort method in the UK [25]. Three serotypes have a correlate of protection well above the 0.35 μg/mL benchmark value: ST-3 (2.83 μg/mL), ST-19F (1.17 μg/mL), and ST-19A (1.00 μg/mL). These three serotypes have been imperfectly controlled by PCV-13 childhood programmes with high uptake rates as shown in Canada [15]. Correlates of protection against nasopharyngeal infection are also much higher than against IPD: 5 μg/mL on average in a study in the USA and there is also a variation according to the serotype [26, 27]. An increase in the incidence of antibody-resistant serotypes in the overall population cannot thus be excluded if a less immunogenic and effective vaccine replaces PCV-13 in children.

## Antimicrobial resistance

Antimicrobial resistance of *Sp* strains seems to be more associated with clonal genotypes than serotypes [28]. For this reason, the proportion of resistant IPD strains within a specific serotype varies with time and geography. At MNL, IPD strains isolated from cultures are tested for resistance to seven drug classes including β-lactams, macrolides, tetracyclines, fluoroquinolones, clindamycin, chloramphenicol, and trimethoprim/sulfamethoxazole. For β-lactams, resistance is tested for penicillin (Clinical and Laboratory Standards Institute oral breakpoint) and ceftriaxone (Clinical and Laboratory Standards Institute meningitis breakpoint). Methods to assess resistance are described in a previous publication [29]. The antimicrobial profile of strains belonging to different serotype groups is shown in Table 1. Note that not all strains submitted to NML are tested. Those originating from LSPQ are not included in the Table as they represent a biased sample of isolates from Quebec.

Among the strains covered by PCV-13, 13% are multiresistant, defined as a resistance to three or more antimicrobial classes. Multiresistant strains in this PCV-13 group mostly belong to serotypes 19A and 9 V. Serotypes 22F and 33F, which are covered by PCV-15, PCV-20, and PCV-21 have a high frequency of monoresistance (59%) but a low frequency of multiresistance (3%). PCV-20 covers 5 serotypes not in PCV-15. Among these, serotypes 11A, 12F, and 15B/C have a high frequency of monoresistance, although multiresistance is less common in this group. Among PCV-21-specific serotypes, three are particularly problematic: 15A (53% monoresistance and 43% multiresistance), 23A (37% monoresistance and 24% multiresistance), and 24F (78% monoresistance and 44% multiresistance).

Antimicrobial resistance is one of the plausible mechanisms that favour the emergence of a particular serotype following the introduction of a new PCV in a childhood programme [30]. Antibiotic resistance may be problematic at the clinical level for the treatment of otitis media mainly [31]. Using the vaccine with the broadest coverage of resistant serotypes for the childhood programme would be preferable, and PCV-20 has an advantage over PCV-15 in this regard.

## Invasiveness

Invasiveness can be defined as the capacity of a particular organism to spread from a primary nasopharyngeal mucosal infection to a systemic or localized secondary infection and can be measured as the number of IPDs per colonization event in a longitudinal study (the attack rate) or as the case-to-carrier ratio combining IPD surveillance and carriage prevalence data [32]. Invasiveness is more related to capsular characteristics defining the serotype than clonal genotypes having different capsular expressions [33].

Two meta-analyses reporting serotype-specific invasiveness indices have been published [34, 35]. The first one was based on four studies published between 2000 and 2015, along with 13 unpublished datasets [34]. Invasiveness was measured as the ratio of IPD incidence to carriage prevalence in children 0–59 months of age in a particular setting using the ST-19A ratio as a reference for the estimation of serotype-specific odds ratios (OR) in a random-effects model. In the other meta-analysis, Bayesian models were applied to 20 datasets in children and five combining data on child carriage and adult IPD [35]. The advantage of this Bayesian methodology over the traditional OR technique is that it provides absolute rather than relative indices, which are more robust for infrequent serotypes, although there was a good level of concordance between results produced by the two methods [35]. For our purpose, absolute invasiveness indices in the second meta-analysis were converted into relative indices using the median ST-17F value as a common reference.

As shown in Table 2, ST 22F has a lower than the median invasiveness value and the ST 33F value is close to the median in the first meta-analysis [34]. The invasiveness ranking of these two serotypes is above average in the second meta-analysis: 18/61 for 22F and 6/61 for 33F [35]. Among PCV-20-non-PCV-15 serotypes, two are among those having the highest invasiveness indices (ST 8 and ST 12F). These observations are important, as both PCV-15 and PCV-20 are authorized for use in children and may induce a replacement involving with highly invasive serotypes not induced in their composition. The use of PCV-15 could thus open the door to the emergence of ST 8 and/or ST 12F but not if PCV-20 is used. It should be noted that serotypes not included in PCV-20 and having high invasiveness are not many: none in the first meta-analysis, ST-22D (4/61), ST-38 (7/61), and ST-10F (8/61) in the second metanalysis [34, 35]. This suggests that PCV-20 has the potential to mitigate serotype replacement more effectively than PCV-15, a hypothesis supported by the results of a dynamic model predicting the impact of a shifting from PCV-13 to PCV-15 or PCV-20 for childhood immunization in the United Kingdom [38].

**Table 2.** Serotype-specific relative invasiveness and virulence indices

	Relative invasiveness and ranking (higher to lower)				Case-fatality ratio and ranking (higher to lower)			
Source	Balsells et al. [34]		Løchen et al. [35]		Demirdal et al. [36]		De Miguel et al. [37]	
Reference for calculations	ST 19A		ST 17F		*Adjusted CFR Odds Ratio = 1 for ST 20*		ST 22F	
	RI	Rank/26	RI	Rank/61	OR	Rank/42	RI	Rank/25
PCV–13 (selection)								
3	1.0	8	1.7	24	1.84	6	1.23	7
19A	1.0	10	4.2	12	1.12	27	0.83	14
19F	0.2	23	1.1	29	1.81	8	1.44	3
PCV–15 non-PCV–13 serotypes								
22F	0.6	12	2.5	18	1.20	24	1.00	13
33F	1.0	10	7.5	6	0.99	31	NA	NA
PCV–20 non-PCV–15 serotypes								
8	2.0	5	9.6	5	0.68	38	0.47	20
10A	0.4	16	1.3	27	1.57	13	0.56	18
11A	NA	NA	0.4	44	2.44	2	1.59	2
12F	5.8	3	16.3	2	0.92	32	0.37	21
15B/C	0.3	20	0.6	38	1.31	21	0.77	16
PCV–21 non-PCV–20 serotypes								
9 N	NA	NA	0.4	43	1.38	19	1.11	10
15A	0.3	19	1.1	30	1.66	11	1.26	4
16F	0.2	22	1.0	33	1.94	5	NA	NA
17F	NA	NA	1.0	32	1.70	10	NA	NA
20	NA	NA	0.7	35	1.00	29	NA	NA
23A	NA	NA	0.3	46	1.53	15	1.22	8
23B	0.1	26	1.0	31	NA	NA	NA	NA
24F	0.7	11	NA	NA	NA	NA	1.01	11
31	NA	NA	0.2	55	2.59	1	1.95	1
35B	0.3	21	0.5	39	1.95	4	1.24	5

NA: Not available. RI: Relative invasiveness. CFR: Case fatality ratio.

15B/C: ST 15B and ST 15C are considered as a single entity.

It should be stressed that this analysis has several limitations. First, there was inter-study variation in the two meta-analyses. Second, confidence intervals were not considered in our ranking. Finally, the invasiveness indices in the two meta-analyses were based on carriage prevalence data and rather than carriage incidence data, which can only be estimated in a longitudinal study with repeated samplings with short intervals, a rarity. Obviously, there is an association between the acquisition (incidence) rate of carriage and the prevalence rate of carriage, but this relationship is somewhat confounded by carriage duration, which has been shown to vary between serotypes [33].

## Virulence

The case-fatality ratio (CFR) is the most easily defined and commonly used marker of *Sp* infection virulence and is a stable serotype-associated property [39, 40]. Serotypes with a high CFR tend to exhibit a high carriage prevalence, low invasiveness, and a thicker capsule in vitro [39]. Two meta-analyses on serotype-specific CFRs have been published and the most comprehensive and recent one was selected [36]. A large case series from Spain covering close to 6000 all-ages IPD cases in 2007–2020 that was not included in the selected meta-analysis was also included in the analysis [37].

As shown in Table 2, ST 22F and ST 33F have close to average CFRs. Among the five PCV-20-non-PCV-15 serotypes, ST 11A is particularly virulent, ranking second in both studies. Virulent serotypes are particularly problematic for adults knowing that CFR is increasing with age [36]. Interestingly, PCV 21 covers 6 out of the 10 most virulent serotypes identified in the meta-analysis (31, 11A, 35B, 16F, 3, and 17F) and also 6 out of the 10 most virulent serotypes in the Spanish case series (31, 11A, 6C thanks to 6A, 3, 23A, and 9 N) [36, 37]. This means that PCV-21 would be particularly useful for protecting adults against virulent infections.

Our analysis has several limitations. First precision of CFR estimates was not taken into account for ranking and confidence intervals were large for serotypes rarely seen before the PCV-13 introduction [36, 37]. Second, there was some heterogeneity in the ranking as suggested by high *I* ^2^ values of several summary estimates in the meta-analysis [36]. Although imperfect, the ranking presented gives an idea of the serotypes that should be controlled through direct or indirect protection of adults.

## Immunogenicity

Pivotal immunogenicity trials in children comparing PCVs were identified in product monographs [2, 3]. Trials were mostly conducted in North America and Western Europe, with measurements of opsonophagocytotic activity geometric mean titres (OPA GMT) selected. The OPA GMT ratio of common serotypes was calculated and the mean of these ratios was interpreted as a proxy of the relative immunogenicity of the vaccines in the comparison [49]. Results are presented in Table 3. It can be seen that PCV7 was more immunogenic than PCV-13 (2 trials) [41, 42], that PCV-13 was more immunogenic than PCV15 (3 trials) [43–45] and PCV-20 (3 trials) [46–48].

**Table 3.** Summary results of immunogenicity studies comparing pneumococcal conjugate vaccines in children and adults

Comparison	Study design	Main findings	Antiboby titre/concentration ratio for common serotypes	References
PCV–13 vs PCV–7 children	Phase 3 RCT in the US: 666 healthy infants received PCV–13 or PCV–7 at ages 2, 4, 6, and 12 –15 months with routine pediatric vaccinations	PCV–13-elicited immunoglobulin G titres non-inferior to those elicited by PCV–7 for the 7 common serotypes, although PCV–13 responses generally lower	Post dose 4 OPA GMT PCV–13:PCV–7 ratio = 0.77	Yeh et al. [41]
	Phase 3 RCT: 605 healthy infants in Germany received PCV–13 or PCV–7 at ages 2, 4, 6, and 12–15 months with routine pediatric vaccinations	PCV–13 and PCV–7 responses showed comparable percent responders achieving 0.35 ?g/mL IgG threshold with 6B exception: 77.5% versus 87.1%, respectively. IgG GMCs and OPA GMTs were generally lower than with PCV–7.	Post dose 4 OPA GMT PCV–13:PCV–7 ratio = 0.87	Kieninger et al. [42]
PCV–15 vs PCV–13 children	Phase 3 RCT in 4 countries including the US: 1720 healthy infants randomized to receive 3 + 1-doses of PCV–15 or PCV–13 with other routine pediatric vaccines at 2, 4, 6, and 12–15 months	PCV–15 met non-inferiority criteria by IgG GMCs for all serotypes at post-dose 3 and 4, except for serotype 6A at post-dose 3. and was superior for ST3	Post dose 4 OPA GMT PCV–15:PCV–13 ratio = 0.75	Lupinacci et al. [43]
	Phase 3 RCT in 9 European countries: 1184 healthy infants randomized to receive a 3 + 1-dose regimen of PCV–15 or PCV–13 with other routine pediatric vaccine at 2, 4, and 12–15 months	PCV–15 met pre-specified non-inferiority criteria for all 13 shared serotypes, based on the difference in proportions of participants with serotype-specific IgG concentrations >0.35 μg/mL	Post dose 3 OPA GMT PCV–15:PCV–13 ratio = 0.79	Martinon-Torres et al. [44]
	Phase 3 RCT in four European countries: 1191 healthy infants randomized to receive 2 + 1-doses of PCV–15 or PCV–13 with other routine pediatric vaccines at 3, 5, and 12 months	PCV–15 met non-inferiority criteria for 13 shared serotypes, based on differences in proportions with serotype-specific IgG >0.35 g/ mL and IgG GMC ratios	Post dose 3 OPA GMT PCV–15:PCV–13 ratio = 0.88	Benfield et al. [45]
PCV–20 vs PCV–13 children	Phase 2 RCT in the USA and Australia: 460 healthy infants randomized to receive 3 + 1-doses of PCV–20 or PCV–13 with other routine pediatric vaccines at 2, 4, 6, and 12–15 months	IgG and opsonophagocytic activity responses elicited by PCV–20 were robust and demonstrated a booster response after dose 4 although PCV–20:PCV–13 antibody concentrations and titres ratios were all below unity.	Post dose 3 OPA GMT PCV–15:PCV–13 ratio = 0.72	Senders et al. [46]
	Phase 3 RCT in the USA: 1997 healthy infants randomized to receive 3 + 1-doses of PCV–20 or PCV–13 with other routine pediatric vaccines at 2, 4, 6, and 12–15 months	IgG GMTs 1 month after dose 4 met non-inferiority criteria for all serotypes although all PCV–20:PCV–13 ratios were below unity.	Post dose 3 mean OPA GMT PCV–20:PCV–13 ratio = 0.93	Senders et al. [47]
	Phase 3 RCT in Europe: 1204 healthy infants randomized to receive 2 + 1-doses of PCV–20 or PCV–13 with other routine pediatric vaccines at 2, 4, and 11–12 months	One month after dose 3, 19/20 serotypes met non-inferiority criteria for IgG GMC, except for serotype 6B. PCV–20:PCV–13 OPA GMT ratios were below unity for 12/13 common serotypes	Post dose 3 mean OPA GMT PCV–20:PCV–13 ratio = 0.76	Korbal et al. [48]
PCV–21 vs PCV–15 children	Indirect comparison of the immunogenicity of PCV–15 and PCV–20 using OPA results from 6 trials.	For most of the common serotypes, OPA responses were better for PCV–7 compared with PCV–13, and better for PCV–13 compared with PCV–15 and PCV–21, whereas responses were almost equivalent in the indirect comparison between PCV–21 and PCV–15.	Post dose 4 (3 + 1) mean PCV–15:PCV–20 OPA GMT ratio = 1.04 Post dose 3 (2 + 1) mean PCV–15:PCV–20 OPA GMT ratio = 1.02 corrected to 1.07^a^	De Wals [49]
PCV–15 vs PCV–13 adults	Phase 3 RCT: 1202 pneumococcal vaccine-naïve adults randomized 1:1 to receive a single dose of PCV–15 or PCV–13; randomization was stratified by age (50–64, 65–74, and ≥ 75 years).	PCV–15 met noninferiority criteria compared to PCV–13 for the 13 shared serotypes. PCV–15 met superiority criteria compared to PCV–13 for serotype 3.	Mean PCV–15:PCV–13 OPA GMT ratio = 0.95	Platt et al. [50]
	Phase 3 RCT: 2333 adults randomized in a 3:3:3:1 ratio to receive a single dose of one of three lots of PCV–15 or PCV–13, stratified by age (50–64, 65–74, ≥75 years).	Serotype-specific OPA GMTs were comparable in the PCV–15 combined lots and PCV–13 groups for the 13 shared serotypes.	Mean PCV–15:PCV–13 OPA GMT ratio = 1.11	Simon et al. [51]
PCV–20 vs PCV–13 adults	Phase 3 RCT: 3009 pneumococcal vaccine-naïve adults ≥60 years randomized to receive a single PCV–20 or PCV–13 dose.	Non-inferiority criteria were met for shared serotypes although OPA GMTs were inferior with PCV–20 compared to PCV–13.	Mean PCV–20:PCV–13 OPA GMT ratio = 0.84	Essink et al. [52]
PCV–21 vs PCV–15 adults	Phase 3 RCT in adults 50 years and older who had received PPS–23 previously: 229 received PCV–21 and 119 received PCV–15.	OPA responses are comparable for the six common serotypes: 3, 6A, 7F, 19A, 22F, and 33F.	Mean PCV–21:PCV–15 OPA GMT ratio = 0.89	Platt [53]
	Phase 3 RCT: 350 pneumococcal vaccine–experienced adults ≥50 years randomized 2:1 to receive a single dose of PCV–21 or PCV–15	PCV–21 elicited comparable immune responses to serotypes shared with PCV15 and higher immune responses to serotypes unique to V116	Mean PCV–21:PCV–15 OPA GMT ratio = 1.08	Scott et al. [54]
PCV–21 vs PCV–20 adults	Phase 3 RCT: 2356 pneumococcal vaccine-naïve adults 50 years and more randomized to receive PCV–21 or PCV–20.	PCV–21:PCV–20 OPA GMT ratio is superior to unity for ST 3, 8, 11A, and 33F, inferior to unity for ST 6A, 10A, 19A, and 22F, and close to unity for ST 7F and 12F.	Mean PCV–21:PCV–20 OPA GMT ratio = 1.09	Platt [53]

a Correction of an error discovered in the Supplementary Table S10 in Martinon-Torres’ manuscript.

There is no head-to-head comparison between PCV-15 and PCV-20 in children. To compare these two vaccines, an indirect comparison was carried out using PCV-13 as a common comparator [49]. Results suggested that PCV-20 and PCV-15 have a similar immunogenicity profile in both 2 + 1 and 3 + 1 schedules, with OPA ratio values close to unity. In this review of immunogenicity trials, the Phase 2 trial comparing PCV-13 to PCV-21 in a 3 + 1 schedule was included for the indirect comparison [46]. Recently, results of the Phase 3 trial comparing PCV-20 to PCV-13 in a 3 + 1 schedule were published and the performance of PCV-20 was somewhat better with a mean PCV-13:PCV-20 OPA GMT ratio of 0.93 instead of 0.72 in the Phase 2 trial [46, 47]. When results of the Phase 3 trial are used for the indirect comparison instead of those of the Phase 2 trial, the average PCV-15:PCV-20 OPA GMT ratio is 0.84, indicating a slightly better performance of PCV-15 compared to PCV-20. However, this has little relevance for Canada, as a 2 + 1 schedule is used for the vast majority of children.

Pivotal immunogenicity trials in adults comparing PCVs with OPA GMT measurements were identified in product monographs and a presentation to the CDC [2, 3, 53]. Trials mostly conducted in North America and Western Europe were selected. As seen in Table 3, the immunogenicity of PCV-15 and PCV-13 for shared serotypes were almost similar with a mean OPA GMT ratio ranging from 0.95 to 1.11 according to studies [50, 51]. In another trial, the mean PCV-20:PCV-13 OPA GMT ratio for shared serotypes was 0.84, suggesting a somewhat lower immunogenicity for the vaccine with an extended valency [52]. In pneumococcal vaccine-experienced adults, two trials compared OPA responses to PCV-21 or PCV-15, and the mean PCV21:PCV15 OPA GMT ratio for the common serotypes was, respectively, 0.89 and 1.08 [53, 54]. In another trial in pneumococcal vaccine naïve adults, the PCV-21: PCV-20 ratio was 1.09 for the 10 shared serotypes [53]. As for children, we do not have a direct comparison between PCV-15 and PCV-20 in adults. In indirect comparisons using PCV13 OPA GMTs as a common denominator, the PCV-20:PCV15 GMT OPA ratio was 0.94 comparing Essink’s and Platt’s results and 0.84 comparing Essink’s and Simon’s results [50–52]. All these results suggest that, in adults, PCV-13 is more immunogenic than PCV20 and that PCV15 and PCV21 are close to PCV13.

It appears that an increase in the number of polysaccharide antigens in pneumococcal conjugate vaccines is associated with a decrease in antibody responses. Our analysis was based on OPA measurements but the same conclusion was drawn in a recent review paper focusing on IgG measurements [55]. Several biological mechanisms have been proposed to explain this phenomenon, a very plausible one being the “carrier-induced epitotic suppression” resulting from a competition between anti-polysaccharide and anti-peptide-carrier responses [56, 57]. Results presented in Table 3 suggest that, in children, PCV-7 is slightly more immunogenic than PCV-13, which is slightly more immunogenic than PCV-15 and PCV-21, with the latter two vaccines being equivalent in a 2 + 1 schedule. In adults, PCV-13 and PCV-15 seem to be equivalent, PCV-20 seems to be slightly less immunogenic than the former two vaccines, and PCV-21 seems to lie between PCV-15 and PCV-21. The clinical significance of these differences is not known in terms of direct protection and its duration, as well as the induction of a herd effect. Nevertheless, the induction of higher functional antibody levels is an asset for any vaccine.

## Synthesis: Strengths and limitations of vaccines

Ideally, a pneumococcal conjugate vaccine should contain a broad range of serotypes covering a high proportion of IPD cases occurring in the entire population, especially serotypes with high invasiveness, high virulence, and high antimicrobial resistance. In addition, an ideal vaccine should induce a strong immune response towards all pneumococcal polysaccharides in its composition leading to a high level of direct protection against IPD and also against nasopharyngeal infection by invasive serotypes. New-generation PCVs do not meet all these criteria.

For children, the choice in Canada is between replacing PCV-13 with either PCV-15 or PCV-20. PCV-20 seems to be by far the strongest candidate: it contains five serotypes not in PCV-15 currently causing 15% of IPD cases in the group less than 5 years of age. Some of these serotypes are highly invasive (8 and 12F particularly) or of high virulence (11A). PCV-20 does not have a significant advantage over PCV-15 in covering *Sp* strains resistant to antimicrobials in Canada. In terms of immunogenicity and their capacity to induce functional OPA antibodies, the two new-generation pediatric PCVs have a similar profile. Both PCV-15 and PCV-20 are slightly less immunogenic than PCV-13 and this may have consequences on the duration of protection and also the maintenance of a herd effect against antibody-resistant serotypes such as 19A and 19F, no herd effect being expected against ST 3 [58]. A major advantage of PCV-20 over PCV-15 for children is the low probability of a negative impact on the IPD risk in adults. Results of a dynamic model calibrated on the epidemiological situation in the United Kingdom indicate that PCV-15 use scenarios would be associated with an overall increase in the incidence of IPD, mainly in adults, (estimated at approximately 7% by the end of a 25-year period in the base-case scenario) whereas PCV-20 scenarios would translate into a sustained reduction in the IPD rate (about 12% in the base-case) [38]. These divergent trends are mainly explained by the relative invasiveness of replacing non-vaccine serotypes compared with vaccine serotypes. This signal of a negative effect cannot be ignored.

For elderly adults, PCV-21 is the strongest candidate, especially if PCV-15 or PCV-20 is used in children, as this would likely lead to an increase in the risk of IPD and also non-invasive pneumococcal pneumonia caused by PCV-21-specific serotypes. Another advantage of PCV-21 over PCV-20 for elderly adults is its capacity to prevent IPD caused by virulent serotypes particularly ST 31 and ST 35B.

For adults at very high risk of IPD (i.e. immunocompromised individuals), a sequential PCV-20 + PCV-21 vaccination would be the most effective schedule. Another advantage of a dual vaccination is the boosting of the response to ST-3 and ST19-A, which have elevated correlates of protection.

Finally, a pneumococcal programme in Canada using only two products, PCV-20 and PCV-21, would be easy to implement and manage.

In Europe, PCV-20 was not authorized in a 2 + 1 schedule for children by the European Medicines Agency based on a lower immune response compared to PCV-13 as measured by IgG concentrations whereas responses were more similar when given as a 3 + 1 regimen [59]. In contrast, PCV-15 was authorized by the European Medicine Agency according to 3 + 1 and 2 + 1 schedules for children [60]. However, when OPA titres are considered, responses to PCV-15 and PCV-20 are very similar both in a 3 + 1 and 2 + 1 schedule although lower than PCV-13 responses by about 25% [49].

## Conclusions

In this manuscript, a new and comprehensive approach to selecting the optimal immunization strategy to control pneumococcal disease is proposed and applied to the Canadian context. This approach coupled with dynamic modelling and economic evaluations including budget impact could be applied to most jurisdictions using PCV-13 or PCV-10 in children according to a 2 + 1, a 3 + 1, or a 1 + 1 schedule with high uptake rates. The off-label use of PCV-20 according to a 2 + 1 schedule as recommended in Canada could be problematic in some jurisdictions but this is another debate.
